# Solitary pulmonary nodule imaging approaches and the role of optical fibre-based technologies

**DOI:** 10.1183/13993003.02537-2020

**Published:** 2021-03-25

**Authors:** Susan Fernandes, Gareth Williams, Elvira Williams, Katjana Ehrlich, James Stone, Neil Finlayson, Mark Bradley, Robert R. Thomson, Ahsan R. Akram, Kevin Dhaliwal

**Affiliations:** 1 Centre for Inflammation Research, Queen's Medical Research Institute, The University of Edinburgh, Edinburgh, UK; 2 Centre for Photonics and Photonic Materials, Dept of Physics, The University of Bath, Bath, UK; 3 Institute for Integrated Micro and Nano Systems, School of Engineering, The University of Edinburgh, Edinburgh, UK; 4 EaStCHEM, School of Chemistry, The University of Edinburgh, Edinburgh, UK; 5 Institute of Photonics and Quantum Sciences, School of Engineering and Physical Sciences, Heriot-Watt University, Edinburgh, UK

## Abstract

Solitary pulmonary nodules (SPNs) are a clinical challenge, given there is no single clinical sign or radiological feature that definitively identifies a benign from a malignant SPN. The early detection of lung cancer has a huge impact on survival outcome. Consequently, there is great interest in the prompt diagnosis, and treatment of malignant SPNs. Current diagnostic pathways involve endobronchial/transthoracic tissue biopsies or radiological surveillance, which can be associated with suboptimal diagnostic yield, healthcare costs and patient anxiety. Cutting-edge technologies are needed to disrupt and improve, existing care pathways. Optical fibre-based techniques, which can be delivered *via* the working channel of a bronchoscope or *via* transthoracic needle, may deliver advanced diagnostic capabilities in patients with SPNs. Optical endomicroscopy, an autofluorescence-based imaging technique, demonstrates abnormal alveolar structure in SPNs *in vivo*. Alternative optical fingerprinting approaches, such as time-resolved fluorescence spectroscopy and fluorescence-lifetime imaging microscopy, have shown promise in discriminating lung cancer from surrounding healthy tissue. Whilst fibre-based Raman spectroscopy has enabled real-time characterisation of SPNs *in vivo*. Fibre-based technologies have the potential to enable *in situ* characterisation and real-time microscopic imaging of SPNs, which could aid immediate treatment decisions in patients with SPNs. This review discusses advances in current imaging modalities for evaluating SPNs, including computed tomography (CT) and positron emission tomography-CT. It explores the emergence of optical fibre-based technologies, and discusses their potential role in patients with SPNs and suspected lung cancer.

## Abstract 2

**Solitary pulmonary nodules are a huge diagnostic challenge. Optical fibre-based technologies, in conjunction with bronchoscopic and transthoracic platforms, are promising novel diagnostic tools in the detection of early lung cancer.** https://bit.ly/3cOIDx4

## Introduction

**FIGURE 1:**
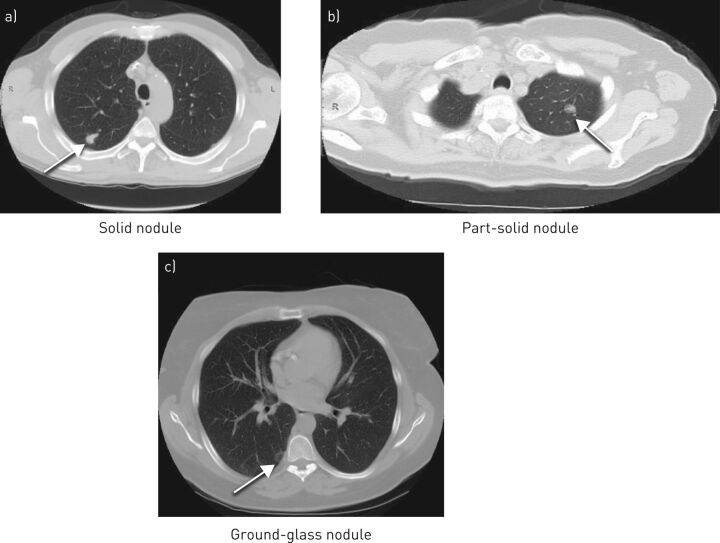
Computed tomography images of different types of solitary pulmonary nodules.

Solitary pulmonary nodules (SPNs) are defined as spherical radiographic opacities, measuring less than 3 cm in diameter, which are surrounded by aerated lung and are not associated with other thoracic abnormalities [1]. They are further sub-classified as solid, part-solid and ground-glass nodules based upon computed tomography (CT) attenuation (figure 1).

The widespread use of CT in clinical practice has made it commonplace to detect nodules incidentally, with a prevalence of 13% in non-screening populations [1]. In screening populations, CT can detect nodules in ∼50% of individuals aged over 50 years with a smoking history [2]. SPNs remain an evolving clinical challenge, which cause clinical and diagnostic uncertainty. Whilst the majority will be benign, some will represent early treatable lung cancer. Lung cancer remains the most common malignancy and the most common cause of cancer related deaths worldwide [3]. Early detection has an impact on survival outcome: the 5-year survival rate in stage I lung cancer is 80%, compared with 10% in stage IV disease [4]. Consequently, there is great interest in the early identification and treatment of malignant SPNs.

Advances in low-dose CT (LDCT) technology have enabled numerous lung cancer screening studies. Collectively, these studies demonstrate a mean nodule prevalence of 33% (17–53%) with a lung cancer prevalence of 1.4% (0.5–2.7%) [1]. The largest screening study, the National Lung Screening Trial, demonstrated a 20% relative reduction in lung cancer mortality with LDCT [5]. The NELSON study has recently shown that LDCT screening led to 24% reduction in 10-year lung cancer mortality in high risk men [6]. The future implementation of national lung cancer screening initiatives [7, 8] will generate increasing numbers of secondary care referrals with SPNs requiring further evaluation.

There is no single clinical sign or radiological feature that definitively identifies malignant SPNs. The British Thoracic Society (BTS) guidelines advocate the use of validated risk prediction models [9, 10], coupled with radiological assessment of nodule size and morphology, to characterise the risk of malignancy [1]. The majority of subcentimetre SPNs undergo radiological surveillance, observing for evidence of growth or the development of sinister features. This results in a significant number of “possible lung cancers” with associated radiation exposure, healthcare costs [11] and patient anxiety [12].

In high-risk SPNs, pre-operative histological confirmation is recommended [1, 13]. Recent advances in bronchoscopic and CT-guided transthoracic approaches mean it is possible to reach the majority of SPNs. Traditionally, CT-guided transthoracic needle biopsy (CT-TNB) has been the preferred approach due to an overall diagnostic yield of >90% [14]. CT-TNB is now extensively used in developed countries [15]. However, availability is limited in low-resource settings due to lack of access to CT equipment and few trained operators [16].

Advanced bronchoscopic platforms, including radial endobronchial ultrasound (rEBUS) and navigational bronchoscopy, have improved the diagnostic yield of bronchoscopy for SPNs [17]. rEBUS is a commonly available bronchoscopic imaging modality [18]. It uses a rotating ultrasound transducer to generate 360° sonographic images of the surrounding lung parenchyma, which enables identification of lesions based on alterations in echogenicity [19]. The rEBUS probe can be delivered within a guide sheath, which serves as an extended working channel, or can be passed *via* an ultrathin bronchoscope [20] to access the subsegmental airways [21]. Consequently, rEBUS has shown good diagnostic performance in the evaluation of distally situated lesions [22]. Navigational bronchoscopy is an emerging endoscopic technique, which enables navigation to hard-to-reach lesions using superimposed reconstructed three-dimensional CT images [23, 24]. Navigational bronchoscopy systems, such as superDimension Navigation System (Medtronic, Minneapolis, MN, USA), are available in over 800 centres worldwide [25]. However, a barrier to more widespread adoption is cost, with system costs of €145 000 and higher overall procedure costs (€2170), compared with CT-TNB (€1510) [24] and rEBUS (€1680) [26]. The major advantage of rEBUS and navigational bronchoscopy is the lower risk of procedure-related complications compared with CT-TNB, such as pneumothorax (3–5% *versus* 23%) [20, 27]. Multiple bronchoscopic techniques (used in combination) deliver a higher diagnostic yield than each method alone [28]. A recent meta-analysis concluded that rEBUS combined with navigational bronchoscopy may be preferable in sampling SPNs >2 cm [29]. This multimodal bronchoscopic approach delivers an acceptable diagnostic yield (>80%) with considerably lower risk of adverse events, compared with CT-TNB. Nevertheless, the negative predictive value of navigational bronchoscopy is only 56% [30], which may be related to sampling inflammatory changes surrounding malignant tissue [31]. Combined navigational bronchoscopy and rEBUS may improve the negative predictive value for malignancy in peripheral lung lesions [32]. However, in many cases a negative biopsy does not provide the clinician with sufficient confidence to discharge the patient from radiological or interventional follow-up.

Fibre-based approaches are compatible with existing bronchoscopic and transthoracic platforms [33–38], and have the potential to augment the diagnostic pathway in SPNs. Optical fibre-based techniques delivered *via* the working channel of a bronchoscope or *via* transthoracic needle, permit high-resolution imaging of the distal lung parenchyma [39]. These novel technologies have the capability to identify target biopsy sites, thereby optimising diagnostic accuracy and avoiding repeated procedures. Optical endomicroscopy (OEM), an autofluorescence-based imaging technique, demonstrates abnormal alveolar structure in SPNs *in vivo* [33–35]. Alternative optical fingerprinting approaches, including time-resolved fluorescence spectroscopy (TRFS), fluorescence-lifetime imaging microscopy (FLIM) and Raman spectroscopy, have shown promise in detecting cancerous lung tissue [40, 41]. These state-of-the-art optical techniques have the capability to streamline the current care pathway by minimising years of CT surveillance and expediting surgical intervention when necessary. Whilst these are not yet in routine clinical use, they have the potential to deliver *in situ* diagnostics, and aid immediate treatment decisions in patients with SPNs.

In this review, we discuss the current imaging modalities for evaluating SPNs, including CT and positron emission tomography-computed tomography (PET-CT), and discuss advances in artificial intelligence approaches and molecular imaging. We review optical fibre-based approaches in lung cancer and explore the role that these emerging technologies may play in the field of SPNs.

## Computed tomography

Multi-detector CT has revolutionised the detection and characterisation of SPNs. The evaluation of CT scan appearances plays a key role in the initial assessment of risk of malignancy in SPNs. In addition, CT is the imaging modality of choice for radiological follow-up of SPNs with low risk of malignancy [1, 13, 42].

Surveillance with serial CT chest imaging is performed in low-risk SPNs, in order to use an assessment of nodule growth to discriminate between benign and malignant SPNs [1]. SPN size has traditionally been assessed by measuring the maximum transverse cross-sectional diameter. However, this approach is limited by poor reliability [43]. Volumetric analysis uses computational algorithms to segment and calculate SPN volumes (expressed as volume doubling time (VDT)). This alternative tool to assess SPN growth has demonstrated improved sensitivity [44, 45] and lower false positive rates [46]. Nodule segmentation performance varies across different software platforms [47, 48]; therefore, measurement standardisation will be essential in facilitating future implementation [1].

Malignant SPNs demonstrate a wide range of VDTs. Therefore, establishing a VDT threshold, which differentiates benign from malignant SPNs is challenging. Sub-solid SPNs, encompassing part-solid and ground-glass nodules, demonstrate a more indolent growth pattern and confer a good prognosis [49]. These require a less interventional management approach compared with solid SPNs [13]. The application of CT surveillance in sub-solid SPNs is difficult, as the presence of indistinct margins and longer VDTs has implications on frequency and duration of follow-up. The observed VDTs for malignant sub-solid SPNs range between 400 and 1100 days [50], and they may grow after prolonged periods of stability [51]. Therefore, the BTS and Fleischner Society guidelines recommend longer total CT follow-up periods for sub-solid SPNs [1, 13], carrying mean costs of ∼€2720 per patient [52]. The advantage of this watchful waiting approach is the avoidance of unnecessary invasive procedures in individuals with benign disease. However, CT surveillance can have a considerable psychological impact in patients with SPNs. The detection of an SPN can adversely impact upon the patient's quality of life [53], causing anxiety [12] and distress [54]. Patients can initially fear they have underlying lung cancer and may carry the burden of diagnostic uncertainty over many years of follow-up [55].

The number of incidental SPNs is expected to rise due to the widespread use of thoracic CT across multiple disciplines in clinical practice. For instance, cardiac CT has been recommended as a first-line investigation in patients presenting with new-onset chest pain [56, 57]. Some CT scanners limit the field of view (excluding the lungs) to optimise coronary artery spatial resolution. However, a number of centres include the full field of view reconstruction of the thorax, which in turn identifies incidental SPNs [58, 59]. The application of advanced computational methods in radiology are likely to play a key role in the assessment of SPNs. Radiomics, the high-throughput extraction of quantitative features from radiographic images, has shown promise in characterising lung tumour phenotypes, including predicting prognosis [60, 61] and response to therapies [62]. This approach may have a role in predicting malignancy in SPNs [63, 64]. There has also been interest in developing CT image analytics, using artificial intelligence approaches, to enable high-throughput identification and assessment of SPNs [65, 66].

Machine learning is a form of artificial intelligence in which computational algorithms learn to recognise patterns in mass data and make accurate predictions with minimal human intervention. The future implementation of national LDCT screening initiatives will likely result in large numbers of additional SPNs requiring CT surveillance, with consequential impact on workforce and CT scanner capacities [65]. There is great interest in using machine learning techniques to detect and risk stratify SPNs [67, 68]. These automated approaches have the potential to reduce radiologists’ workload by avoiding unnecessary follow-up in benign SPNs and enabling earlier identification of malignant SPNs [69]. Researchers at Google designed a deep learning algorithm, which was retrospectively applied to ∼42 000 lung cancer screening CT scans. They demonstrated that their model performed as well as or better than radiologists in detecting malignant SPNs [70]. Baldwin *et al.* [65] applied a machine learning prediction model (using radiological information alone) to assess the risk of malignancy in small SPNs, and demonstrated a sensitivity of 99.5%, outperforming the Brock model risk calculator [9]. Whilst these computer-aided decision supporting technologies hold promise in the field of SPNs, there are number of challenges, including patient selection bias [71], accountability and data privacy issues [72].

## Positron emission tomography-computed tomography imaging

Positron emission tomography (PET) with ^18^F-fluorodeoxyglucose (^18^F-FDG), an analogue of glucose, provides functional information based on the increased rates of glucose uptake in cancer cells. Integrated PET-CT scanners offer a synergistic combination of anatomical and metabolic imaging, maintaining the sensitivity of CT and specificity of PET [73]. This imaging modality is commonly used in the diagnosis and staging of lung cancer [74], and now has a key role in the management of SPNs [1]. However, the availability of PET-CT greatly varies throughout the world. For instance there are 6.5 scanners per million people in the USA [75], whereas >90% lower-middle income countries do not have access to PET-CT facilities [76]. This is primarily due to high equipment costs (including annual service costs of ∼€864 000) [77], and limited availability of trained personnel [78].

Current guidelines advocate ^18^F-FDG PET-CT in the further evaluation of high-risk solid SPNs [1, 13]. Herder *et al.* [10] demonstrated that incorporating a qualitative measure of ^18^F-FDG avidity in an existing validated risk prediction model resulted in improved diagnostic accuracy in SPNs. The addition of ^18^F-FDG PET information increased the area under the curve by 13% (0.79 to 0.92) [10]. However, this imaging modality has limitations. The pre-test probability of malignancy influences interpretation of PET-CT, with high-risk individuals at risk of false-negative results [42]. Furthermore, the use of ^18^F-FDG PET-CT in subcentimetre SPNs has been unclear, due to limitations of PET-CT spatial resolution [1]. Advances in image reconstruction technologies may enable more accurate PET-CT characterisation of subcentimetre SPNs, in which assessment of metabolic activity is challenging [79].

Image reconstruction methodologies have an impact on the measurement of standardised uptake value (SUV; a relative measure of FDG uptake) in SPNs [80]. The most widely used algorithm, ordered subset expectation maximisation (OSEM), generates a reconstructed PET-CT image from the raw data through successive approximations. However, image noise increases with each iteration, which often results in an underestimation of SUV [81]. Advances in PET-CT image reconstruction algorithms, such as Q.Clear (GE Healthcare, Chicago, IL, USA), demonstrate enhanced image quality and improved quantification accuracy, particularly in small SPNs [82]. Furthermore, the implementation of Q.Clear in conjunction with artificial intelligence techniques improves the detection of SPNs in PET-CT, compared with conventional OSEM [83]. Q.Clear yields significantly elevated maximum SUV values in SPNs [82], although this does not alter the diagnostic performance of the Herder model [84].

^18^F-FDG is the most commonly used radiotracer in PET-CT imaging. However, it is not cancer specific, and false-positive uptake is seen in inflammatory or infective conditions [85], which contributes to benign resection rates (12–15%) [86, 87]. Consequently, there have been attempts at developing other PET-CT molecular imaging approaches for SPNs. Scafoglio *et al.* [88] demonstrated that the activity of the sodium-glucose transporter 2, a potential marker of metabolically active early-stage lung adenocarcinoma, could be imaged *in vivo* using methyl 4-deoxy-4-[^18^F] fluoro-alpha-D-glucopyranoside (Me4FDG) PET-CT. Non-approved radiotracers, such as ^18^F-fluorodeoxythymidine (a tissue proliferation tracer) and ^11^C-methionine (a protein metabolism tracer), have also been investigated [89]. However, the potential of these alternative radiotracers in SPNs remains unclear. For example, a meta-analysis demonstrated ^18^F-fluorodeoxythymidine PET was less sensitive than ^18^F-FDG PET in differentiating benign and malignant lesions [90]. Furthermore, the short half-life of ^11^C (20 min), restricts the use of ^11^C-methionine PET to centres with an on-site cyclotron and radiochemistry facilities.

## Optical endomicroscopy

Advanced bronchoscopic techniques, such as rEBUS and navigational bronchoscopy [91, 92], and robotic platforms [93] enable access to most of the lung parenchyma *via* endobronchial means. This can be further supplemented by transthoracic approaches [94]. However, the diagnostic yield of transbronchial and CT-guided biopsies for small SPNs is influenced by nodule size [29]. Optical endomicroscopy (OEM) enables visualisation of the distal lung parenchyma at high-resolution. Therefore, this novel approach may have a role in augmenting the diagnostic accuracy of existing sampling techniques, such as rEBUS, in the field of SPNs [33, 34].

There has been great interest in translating confocal microscopy techniques to enable endomicroscopic exploration of the respiratory system [95]. Fibre-based confocal fluorescence microscopy is an OEM technique, in which the microscope objective luminates a flexible fibre-optic, containing thousands of light-guiding cores, using a laser-scanning unit to scan across the fibre bundle. Each core in the fibre bundle acts as a single pixel in the resulting image with emitted fluorescence travelling back up the same core to a detector [96]. The most commonly used system for pulmonary imaging has a depth of focus of 0–50 μm, circular 600 μm field of view and scan rate of 12 frames·second^−1^ (Alveoflex, Cellvizio; Mauna Kea Technologies, Paris, France) [97]. The flexibility of the fibre, coupled with the imaging speed, enables real-time microscopic imaging of the distal lung.

OEM can be easily performed during bronchoscopy, with the fibre introduced *via* the working channel. This provides the clinician with real-time endomicroscopic imaging of the respiratory tract, thereby extending the field of interventional pulmonology to the distal lung and the cellular level [39]. This minimally invasive technique, which adds approximately 10 min to a conventional bronchoscopy procedure, is well tolerated in topically anaesthetised spontaneously breathing patients [39, 97]. It has a good safety profile and has been studied in numerous respiratory diseases in multiple centres worldwide [34, 35, 98, 99].

**FIGURE 2:**
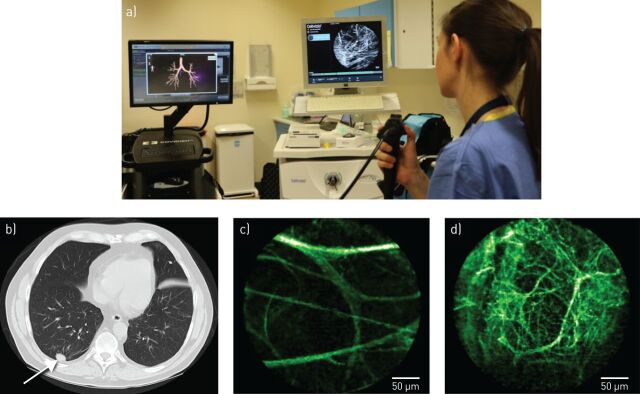
a) The delivery of fibre-based optical endomicroscopy (OEM), in conjunction with navigational bronchoscopy, to access a distal solitary pulmonary nodule (SPN) in the clinical setting. Computed tomography (CT) and OEM images from an individual patient presenting with an SPN: b) CT demonstrating solid SPN; c) OEM image demonstrating surrounding healthy elastin structure; d) OEM image demonstrating distorted, abnormal elastin structure within the SPN (subsequently confirmed as benign on histopathological analysis) [35].

At 488 nm excitation, OEM generates microscopic images of human alveolar structure through the autofluorescence of elastin [39], which represents 50% of peripheral lung connective tissue fibres [100], and is unaffected by collagen fluorescence [101]. In health, the elastin fibre framework appears as a network of linear contours encircling alveolar ducts and surrounding extra-alveolar micro-vessels. However, this structure can be distorted in distal lung pathologies [102], including SPNs [35] (figure 2).

Label-free OEM pulmonary imaging is an attractive modality for distinguishing normal from abnormal lung tissue. Visual analysis of OEM images in pathological cases demonstrates distortion of the alveolar network, characterised by a tangle of elastin fibres. Advanced image analysis techniques may aid clinicians by enabling automated image classification for the diagnosis of distal lung pathologies [103–105]. Hébert *et al.* [106] analysed OEM images from the distal lung in healthy volunteers and patients with interstitial lung disease. They discriminated healthy alveolar structure from pathological in 86.3% and 95.1% of non-smokers and current/ex-smokers, respectively. Rakotomamonjy *et al.* [107] explored the feasibility of lung cancer detection using OEM with machine learning tools, in patients with bronchial squamous cell carcinoma and healthy volunteers. The authors demonstrated that the application of topical methylene blue (a contrast agent) in conjunction with a 660 nm OEM system had 90% classification accuracy in the diagnosis of lung cancer. Whilst these are encouraging studies, they all used very limited datasets. Thus, with larger data accrual, automated computational analysis of OEM images may have a role in the further evaluation of SPNs [108].

Fibre-based OEM, in conjunction with rEBUS and/or navigational bronchoscopy, can access the vast majority of SPNs, including subcentimetre, peripherally located lesions [33–35]. This enables high-resolution imaging of SPNs, including the nodule penumbra which is a key area to sample as intratumoral necrosis is often present [109]. Wijmans *et al.* [37] demonstrated that fibre-based OEM, delivered *via* transthoracic approach, enabled real-time visualisation of pleural abnormalities in mesothelioma, which could be distinguished from benign pleural disease. Fibre-based OEM has the potential to aid identification of optimal biopsy sites, thereby increasing the diagnostic yield and avoiding repeated conventional sampling procedures with associated complications [33–38].

Label-free fibre-based OEM shows benign and malignant SPNs are indistinguishable *in vivo*, as they both demonstrate abnormal alveolar fluorescence images [35, 110]. Seth *et al.* [35] evaluated the efficacy of incorporating additional information from OEM in the assessment of risk of malignancy in SPNs (including 66 benign and 25 malignant SPNs). They demonstrated that there were no features, obtained through either manual assessment or automated feature extraction, that significantly improved the operator characteristics of existing SPN risk calculators. The use of contrast agents or fluorescent probes in OEM may play a key role in enabling detailed assessment of risk of malignancy in SPNs.

Exogenous fluorescent contrast agents, such as intravenous fluorescein and topical acriflavine, have been used to enhance bronchial vascular and cellular structure imaging in pulmonary OEM [111–113]. Fluorescein-aided fibre-based OEM enables *in vivo* imaging of inflammatory and cancer cells in the distal lung parenchyma [114], by enhancing fluorescence of the stromal background [115]. Wijmans *et al.* [36] demonstrated the use of an endosonography-guided needle-based OEM system, in conjunction with systemically administered fluorescein, to assess lung cancers and mediastinal lymph nodes. They showed that this needle-based approach enables real-time *in vivo* visualisation of malignant cells in ∼90% of patients with lung cancer (including stage I–IV non-small cell and limited disease small cell lung cancers) [36]. Whilst fluorescein is a safe and widely available contrast agent, one limitation is that it does not stain central airway bronchial epithelial cells [114]. Acriflavine is an alternative fluorescent dye, which strongly labels cell nuclei and superficial epithelial cells. The use of topical acriflavine, in conjunction with fibre-based OEM, has demonstrated very high sensitivity and specificity in detecting malignant endobronchial lesions (including non-small cell and small cell lung cancers, and non-neoplastic lesions, such as sarcoidosis) [113]. However, there has been concern regarding the potential carcinogenic effects of acriflavine [116], which has limited further uptake of this technique. Methylene blue is a non-toxic exogenous fluorophore, which enables direct visualisation of cell nuclei, and can be safely used topically to image pre-cancerous lesions *in vivo* [116]. Methylene blue-aided OEM permits cellular imaging of bronchial cancer [107] and distally situated SPNs [33]. However, in order to give a fluorescence signal, methylene-blue requires a 660 nm excitation wavelength OEM system. Finally, there has been interest in developing Smartprobes, bespoke compounds which emit fluorescence following activation by specific signalling molecules and proteins, to detect lung pathologies [117–119]. The potential of fluorescein-based Smartprobe imaging in lung cancer has been demonstrated by targeting epidermal growth factor receptor mutations in cell line xenograft mouse model [120] and in *ex vivo* human lung cancer tissue for visualising matrix activity [118]. Targeted OEM imaging with 488 nm compatible, topically administered Smartprobes has now commenced in clinical cohorts with confirmed or suspected lung cancer (ClinicalTrials.gov, NCT02676050). The integration of alternative approaches [118, 121] and multiplexed molecular imaging [122] makes Smartprobe OEM a promising technique for evaluating malignancy in SPNs.

OEM is an attractive translational technology, which has the potential to augment the diagnostic pathway in SPNs. In the context of pulmonary disease, the FIVE2 device (OptiScan Imaging Ltd, Melbourne, Australia) has been developed for pre-clinical research. This rigid, hand-held instrument, which requires delivery *via* the transthoracic approach, has been used to evaluate malignant pleural disease *in vivo* [123]. There is a commercially available flexible fibre-based OEM system (Cellvizio; Mauna Kea Technologies, Paris, France), which is used as a standard diagnostic modality in gastroenterology [124]. It has also received US Food and Drug Administration approval for use with existing endobronchial platforms and transthoracic biopsy needles [125]. The initial OEM system costs are ∼€88 000 [126], and each fibre costs ∼€6000; however, these can be reprocessed for use in up to 20 examinations in multiple patients [107]. This technology has a growing installed user-base (>600 systems worldwide) [127], especially following the introduction of reimbursements for use in gastro-intestinal procedures [124]. The development of single-use OEM fibres [128], and low cost modular imaging systems [122] may herald the wider adoption of fibre-based techniques in the field of interventional pulmonology. Whilst OEM alone aids accurate localisation of SPNs, the addition of novel spectroscopic techniques has the potential to enable identification of malignant features in SPNs.

## Time-resolved fluorescence spectroscopy and fluorescence-lifetime imaging microscopy

Advanced spectroscopic technologies, such as time-resolved fluorescence spectroscopy (TRFS) and fluorescence-lifetime imaging microscopy (FLIM), provide quantifiable biochemical information, which may aid the identification of malignant tissue [129]. These functional methodologies can give a direct insight into the molecular interactions of a fluorophore within its biological environment [130]. Fluorescence intensity, the primary output of OEM, is particularly challenging to quantitively measure in the clinical setting, due to tissue movement [131]. In contrast, fluorescence lifetime, defined as the time a fluorophore spends in excited state before returning to ground state by emitting a photon, is largely independent of external factors [132].

Time-resolved fluorescence spectroscopy techniques detect emitted photons following sample excitation by a pulsed laser source, thereby allowing a lifetime to be calculated for each pixel in a field of view. FLIM delivers highly spatially resolved images (from the contrast in fluorescence lifetimes) to enable the rapid localisation of detected abnormal tissue [133]. To date, time-resolved fluorescence techniques have been relatively slow, limiting clinical implementation. However, this has been overcome by advances in sensor technology [134–136], which break the usual compromise between time-resolution and processing rates, enabling high sensitivity optical fingerprinting. Therefore, there is great interest in clinically translating time-resolved fluorescence techniques for cancer detection [137, 138].

Altered metabolism is a hallmark feature of cancer cells. This is characterised by the preferential switch from oxidative phosphorylation to aerobic glycolysis (known as the Warburg effect) [139], which favours tumour growth and proliferation [140]. Metabolic co-factors, such as NADH and FAD, are intrinsically fluorescent and spectrally distinct [132]. Therefore, TRFS and FLIM may enable label-free detection of metabolic changes associated with cancer. Skala *et al.* [141] used two-photon FLIM to study NADH and FAD in an *in vivo* model of epithelial pre-cancer. They demonstrated that the fluorescence lifetime of NADH decreases in cancerous tissue. More recently, fluorescence lifetimes of NADH and FAD in human non-small cell lung carcinoma cells have been shown to be significantly shorter than in normal cells using FLIM [142]. The biochemical basis for these variations is unclear. However, Blacker *et al.* [143] demonstrated that changes between oxidative and glycolytic metabolism *in vitro* did not affect NAD(P)H fluorescence decay rates. The lifetime changes observed in cancers may reflect shifts in NADPH/NADH balance.

Label-free fluorescence spectroscopy and imaging have been recognised as potential tools for the detection of pre-cancer/cancer in *ex vivo* human tissue with high specificity [144]. FLIM endoscopy has been used to identify oral pre-cancerous and cancerous lesions, (such as epithelial dysplasia and squamous cell carcinoma) from benign inflammatory conditions *in vivo* [145]. Furthermore, Wang *et al.* [40] investigated the ability of FLIM to differentiate lung cancer from healthy tissue (5 cm from tumour margin) in *ex vivo* human lung specimens. They demonstrated that the fluorescence lifetime of cancerous lung tissues is consistently lower than normal tissues, which they apportioned to the decrease in both NADH and FAD lifetimes. They reported excellent sensitivity and specificity for the detection of lung cancer (92.9% and 92.3% respectively) using FLIM. Therefore, this novel spectroscopic technique may have a role in the detection of lung cancer, in particular the evaluation of risk of malignancy in SPNs.

Commercially available FLIM devices, such as DermaInspect and MPTflex Multiphoton Laser Tomography (JenLab GmbH, Berlin, Germany), have been used to study superficial tumours *in vivo*, such as malignant melanoma [146] and glioblastoma (during craniotomy) [147]. However, these microscope systems are not deliverable to deeper sited tissues, such as the lungs. Kennedy *et al.* [148] have demonstrated confocal FLIM endomicroscopy by incorporating FLIM with Cellvizio fibre-based OEM system, which enabled label-free, real-time imaging of live cells using 488 nm excitation. Thus, fibre-based dual fingerprinting techniques have the potential to provide new diagnostic imaging modalities for SPNs, with the aim of enabling real-time verification of malignant SPNs (supplementary video).

## Raman Spectroscopy

Raman spectroscopy is a label-free optical technique, based on the inelastic scattering of light. Raman spectra contain information on the vibrational and rotational energy transitions of molecules [149], thereby yielding a unique optical fingerprint of the molecular composition of a biological sample, including *ex vivo* lung cancer [150].

Raman spectroscopy is a promising technique for detecting lung cancer *in vivo* [151] due to its ability to detect biochemical changes associated with malignancy, such as higher metabolic activity and changes in lipid and protein levels [152]. Identifying changes in Raman spectra using visual observation can be challenging. However, the recent introduction of machine learning approaches in Raman spectroscopy has demonstrated excellent accuracy in differentiating lung cancer, from healthy lung tissue *ex vivo* [153]. To date, the clinical translation of Raman technology has been limited. Raman scattering is an inherently weak process as only a very small proportion of photons are inelastically scattered. Thus, high intensity illumination and long acquisition times are usually required [154]. Recently, McGregor *et al.* [41] demonstrated the application of a real-time endoscopic Raman spectroscopy system in evaluating normal, inflamed, dysplastic and malignant bronchial tissue (confirmed histologically) *in vivo*. They reported excellent sensitivity (90%) in detecting early lung malignancies. The Raman signatures of inflamed bronchial tissue were also considerably different, compared with other pathologies, thereby highlighting the potential role of Raman spectroscopy in the characterisation of SPNs, the vast majority of which are benign.

**FIGURE 3:**
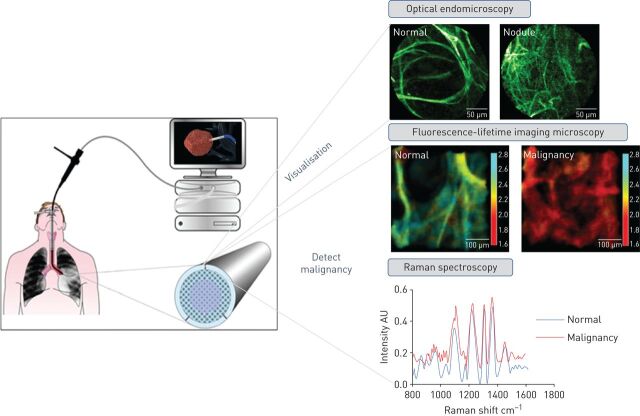
Overview of optical fibre-based technologies, delivered *via* the working channel of a bronchoscope to access and characterise a solitary pulmonary nodule.

The development of a miniature Raman probe, delivered *via* the working channel of a bronchoscope, has enabled acquisition of *in vivo* Raman spectra from the peripheral lung, including normal tissue and SPNs [155]. Furthermore, the development of fibre-based multimodal platforms is underway [156, 157], with the aim of enabling real-time diagnostic imaging and spectroscopic identification of malignant SPNs *in situ* (figure 3).

## Conclusion

SPNs are a clinical and diagnostic challenge. Whilst the majority are benign, some represent early treatable lung cancer. Existing care pathways involve endobronchial/transthoracic tissue biopsies or radiological surveillance, which can be associated with suboptimal diagnostic yield, procedure-related complications and patient anxiety. Recent advances in artificial intelligence approaches in CT and PET-CT hold promise in enabling accurate characterisation of SPNs. Fibre-based optical fingerprinting approaches, in conjunction with endobronchial and transthoracic platforms, may facilitate advanced diagnostic capabilities in patients presenting with SPNs. Autofluorescence OEM has demonstrated aberrant alveolar fluorescence structure in SPNs *in vivo*. Furthermore, novel spectroscopic approaches, such as TRFS, FLIM and Raman spectroscopy, have shown promise in distinguishing malignancy from normal human lung tissue. Fibre-based technologies have the potential to enable *in situ* characterisation and real-time microscopic imaging of SPNs, thereby streamlining the diagnostic pathway by identifying individuals with malignant SPNs early and expediting curative treatment.

## Supplementary material

10.1183/13993003.02537-2020.Supp1 **Please note:** supplementary material is not edited by the Editorial Office, and is uploaded as it has been supplied by the author.
Supplementary video ERJ-02537-2020.SUPPLEMENT

## Shareable PDF

10.1183/13993003.02537-2020.Shareable1 This one-page PDF can be shared freely online.
Shareable PDF ERJ-02537-2020.Shareable
