# Supplementary material for: Solitary pulmonary nodule imaging approaches and the role of optical fibre-based technologies
Source: Eur Respir J. 2021 Mar 25;57(3):2002537. doi: 10.1183/13993003.02537-2020 (PMC8174723; doi:10.1183/13993003.02537-2020)
Supplement: Supplementary file 2 [file ERJ-02537-2020.Shareable.pdf]

SHAREABLE PDF

Solitary pulmonary nodule imaging approaches and the role of optical fibre-based technologies

Susan Fernandes [1], Gareth Williams [1], Elvira Williams [1], Katjana Ehrlich [1], James Stone [1,2], Neil Finlayson [1,3], Mark Bradley [1,4], Robert R. Thomson [1,5], Ahsan R. Akram [1] and Kevin Dhaliwal [1]

Affiliations: [1]Centre for Inflammation Research, Queen's Medical Research Institute, The University of Edinburgh, Edinburgh, UK. [2]Centre for Photonics and Photonic Materials, Dept of Physics, The University of Bath, Bath, UK. [3]Institute for Integrated Micro and Nano Systems, School of Engineering, The University of Edinburgh, Edinburgh, UK. [4]EaStCHEM, School of Chemistry, The University of Edinburgh, Edinburgh, UK. [5]Institute of Photonics and Quantum Sciences, School of Engineering and Physical Sciences, Heriot-Watt University, Edinburgh, UK.

Correspondence: Susan Fernandes, Centre for Inflammation Research, Queen's Medical Research Institute, The University of Edinburgh, 47 Little France Crescent, Edinburgh, EH16 4TJ, UK. E-mail: susan.fernandes@ed.ac.uk

@ERSpublications
Solitary pulmonary nodules are a huge diagnostic challenge. Optical fibre-based technologies, in conjunction with bronchoscopic and transthoracic platforms, are promising novel diagnostic tools in the detection of early lung cancer. https://bit.ly/3cOIDx4

Cite this article as: Fernandes S, Williams G, Williams E, *et al.* Solitary pulmonary nodule imaging approaches and the role of optical fibre-based technologies. *Eur Respir J* 2021; 57: 2002537 [https://doi.org/10.1183/13993003.02537-2020].

This single-page version can be shared freely online.

ABSTRACT Solitary pulmonary nodules (SPNs) are a clinical challenge, given there is no single clinical sign or radiological feature that definitively identifies a benign from a malignant SPN. The early detection of lung cancer has a huge impact on survival outcome. Consequently, there is great interest in the prompt diagnosis, and treatment of malignant SPNs. Current diagnostic pathways involve endobronchial/transthoracic tissue biopsies or radiological surveillance, which can be associated with suboptimal diagnostic yield, healthcare costs and patient anxiety. Cutting-edge technologies are needed to disrupt and improve, existing care pathways. Optical fibre-based techniques, which can be delivered *via* the working channel of a bronchoscope or *via* transthoracic needle, may deliver advanced diagnostic capabilities in patients with SPNs. Optical endomicroscopy, an autofluorescence-based imaging technique, demonstrates abnormal alveolar structure in SPNs *in vivo*. Alternative optical fingerprinting approaches, such as time-resolved fluorescence spectroscopy and fluorescence-lifetime imaging microscopy, have shown promise in discriminating lung cancer from surrounding healthy tissue. Whilst fibre-based Raman spectroscopy has enabled real-time characterisation of SPNs *in vivo*. Fibre-based technologies have the potential to enable *in situ* characterisation and real-time microscopic imaging of SPNs, which could aid immediate treatment decisions in patients with SPNs. This review discusses advances in current imaging modalities for evaluating SPNs, including computed tomography (CT) and positron emission tomography-CT. It explores the emergence of optical fibre-based technologies, and discusses their potential role in patients with SPNs and suspected lung cancer.

Copyright ©ERS 2021. This article is open access and distributed under the terms of the Creative Commons Attribution Licence 4.0.
